# Quality of Life in the Danish Fontan Population is Unchanged Over the Past Decade—A Nationwide Longitudinal Study

**DOI:** 10.1007/s00246-023-03347-y

**Published:** 2023-12-13

**Authors:** Maren Ravndal, Benjamin Kelly, Ola Ekholm, Helle Andersen, Dorte Guldbrand Nielsen, Lars Idorn, Vibeke Hjortdal

**Affiliations:** 1 Department of Cardiothoracic Surgery, Copenhagen University Hospital Rigshospitalet, Blegdamsvej 9, 2100 Copenhagen, Denmark; 2 Department of Cardiothoracic and Vascular Surgery, Aarhus University Hospital, Aarhus, Denmark; 3 National Institute of Public Health, University of Southern Denmark, Odense, Denmark; 4 Department of Pediatrics, Odense University Hospital, Odense, Denmark; 5 Department of Cardiology, Aarhus University Hospital, Aarhus, Denmark; 6 Department of Pediatrics and Adolescent Medicine, Copenhagen University Hospital, Copenhagen, Denmark

**Keywords:** Congenital heart disease, Fontan circulation, Health-related quality of life (HRQoL)

## Abstract

Knowledge about health-related quality of life (HRQoL) over time in Fontan patients is sparse. We aimed to describe HRQoL over a ten-year period in a population-based Fontan cohort. Further, we compared HRQoL in Fontan patients with the general population. In 2011, Danish Fontan patients were invited to participate in a nationwide study assessing HRQoL. Depending on age, 152 participants filled out either the Pediatric Quality of Life Inventory or the 36-Item Short Form Health Survey. After a decade, patients from the initial study were invited to participate in a follow-up study. All were given the same questionnaire as in the first study, plus the 12-Item Short Form Health Survey (SF-12) as part of the Danish National Health Survey. HRQoL over time was described, and SF-12 scores were compared with the general population. A total of 109 Fontan patients completed the questionnaires in both studies. The mean patient age was 14.9 ± 6.6 years and 25.6 ± 6.5 years respectively. Despite an increase in complications, HRQoL did not decrease during the study period. Physical HRQoL scores were lower than mental HRQoL scores at both time points. The SF-12 physical component score was significantly lower in Fontan patients than in the general population (median score 52 vs. 56, *p* < 0.001), while the SF-12 mental component score was comparable (median score 51 vs. 50, *p* = 0.019). HRQoL remained stable over a ten-year period in a contemporary Danish Fontan cohort. Still, the physical HRQoL remained significantly lower than that of the general population.

**Supplementary Information:**

The online version contains supplementary material available at 10.1007/s00246-023-03347-y.

## Introduction

It is over fifty years since the first Fontan procedure was performed, and life expectancy for children born with univentricular hearts is higher than ever before [1]. Although still a palliative procedure, refinements of the surgical and medical care leading to a Fontan circulation have resulted in optimistic survival rates – with an expected post-Fontan 25-year survival rate exceeding 80% [2]. Despite increased survival rates, the morbidity is still high in Fontan patients. Common Fontan-related complications include arrhythmias, impaired exercise capacity, thromboembolism, and Fontan-associated liver disease [3–5]. With as many as half of the patients experiencing complications before even reaching adulthood [6], the health-related quality of life (HRQoL) may be affected. Several cross-sectional studies have investigated HRQoL in Fontan patients, but most studies included younger patients or patients from earlier eras [7–10]. There is limited longitudinal data on HRQoL in Fontan patients [7, 11]. To provide optimal follow-up for Fontan patients, it is crucial to gain a better understanding of how HRQoL evolves over time, as these patients grow up.

In this study, we aimed to describe the development of HRQoL over a ten-year period in a population-based Danish Fontan cohort. We also wanted to compare HRQoL between the Danish Fontan patients and the general Danish population.

## Methods

### Patients

In 2011, 158 out of 179 eligible Danish Fontan patients were included in a nationwide Fontan study investigating HRQoL (Study I). Methods for Study I are previously described [12]. In 2021, the Danish Fontan patients were assessed in a new national study (Study II). Only patients who participated in Study I were considered eligible for Study II. The patients were recruited through the outpatient clinics. All patients gave written consent before inclusion. The protocol met the ethical principles outlined in the 1975 Declaration of Helsinki and received approval from the National Scientific Ethics Committee (H-20028226).

### Quality of Life Questionnaires

In Study I, the patients completed different HRQoL- questionnaires depending on age: children aged 17 or younger filled out the Pediatric Quality of Life Inventory (PedsQL) [13], while patients aged 18 years or older filled out the 36-Item Short Form Health Survey (SF-36) [14]. Patients aged 16–17 years completed both PedsQL and SF-36 in Study I. In Study II, the participants answered the same HRQoL-questionnaire as in Study I. For those who completed both PedsQL and SF-36 in Study I, PedsQL was chosen as the preferred follow-up questionnaire in Study II. Thus, patients aged 17 or younger at the time of Study I, answered the PedsQL in both studies—and patients aged 18 or older at the time of Study I, answered the SF-36 in both studies. The questionnaires were completed either online or on paper in relation to a scheduled outpatient control. Both PedsQL and SF-36 have been uses to assess HRQoL in Fontan patients in other studies, and a 2016 study by Uzark et al. demonstrated significant correlations between PedsQL scores and SF-36 scores within corresponding subscales [15].

### PedsQL

The PedsQL Generic Core Scales Version 4.0 was used for Study I and II. PedsQL is a 23-item questionnaire designed to measure HRQoL [13]. There are several versions available for different age groups, with self-reports available for children, teenagers, and adults. All versions are similar, and the scores are calculated in the same way across the different age versions. The scores can be categorized into four multidimensional subscales: Physical Functioning, Emotional Functioning, Social Functioning, and School Functioning. A Psychosocial Summary Score (a sum of Emotional, Social, and School Functioning) and a Total Score (a sum of all the items) are also calculated. The scores range from 0 to 100, where higher scores indicate better HRQoL.

### SF-36

SF-36 is a 36-item questionnaire made to measure HRQoL in adults. The items can be grouped into eight subscales [14]. Physical Health comprises four subscales (Physical Functioning, Physical Role Functioning, Bodily Pain, General Health), and Mental Health comprise four subscales (Vitality, Social Functioning, Role-Emotional Functioning, and Mental Health). A Physical component score (PCS-36) and a Mental component score (MCS-36) can also be calculated, based on the eight subscales. The PCS-36 and MCS-36 are calculated using a T-score transformation based on a U.S. population norm. The PCS and MCS transformations are standardized to give a mean score of 50 in a population similar to the US norm, potentially causing some score discrepancies when compared to the other SF-36 subscales [14]. For all eight subscales, PCS-36, and MCS-36, higher scores indicate better HRQoL.

### The Danish National Health Survey

In addition to PedsQL or SF-36, the patients were given a second questionnaire in Study II. This included questions from the Danish National Health Survey (DNHS) in 2017. The DNHS is sent out every four years to a random sample of the Danish population. It aims to monitor the national trend in health, morbidity, and health behaviour. The DNHS 2017 consisted of 51 questions on health and 9 questions regarding demographics (sex, age, educational level, etc.). Out of these 51 questions, 22 were regarded relevant for the Fontan population and selected for comparison. These questions were related to HRQoL (12-Item Short Form Health Survey), perceived stress (Cohens perceived stress scale [16]), social relations (frequency of contact with family and friends), physical activity (minutes per week spent in moderate/vigorous physical activity), sociodemographic factors (educational level, employment status), alcohol intake, and smoking behavior. As the 12-Item Short Form Health Survey (SF-12) is a part of the DNHS, eight HRQoL subscales could be calculated, corresponding to the SF-36 subscales; Physical Functioning, Physical Role Functioning, Bodily Pain, General Health, Vitality, Social Functioning, Role-Emotional Functioning, and Mental Health. In addition, a physical component score (PCS-12) and a mental component score (MCS-12) could be calculated. The PCS-12 and MCS-12 scores have been proved accurate compared to the PCS-36 and MCS-36 scores, however, less accuracy is reported when comparing scores in the eight subscales of SF-36 and SF-12 [17]. Of the 183,372 respondents who answered the DNHS in 2017 [18], a random sample of 35,000 persons within the same age range as the Fontan patients were drawn for use as control data. Respondents with missing data on HRQoL and physical activity were excluded. Each Fontan patient was matched with individuals from the general population of the same sex and age.

### Inclusion and Exclusion Criteria

Inclusion criteria were all patients who had completed either the PedsQL or the SF-36 in Study I, and who were still alive with a Fontan circulation at the time of Study II. Patients who did not complete both questionnaires in Study II (PedsQL/SF-36 and DNHS) were also excluded.

### Statistical Analyses

Based on distribution, data are described as mean ± standard deviation (SD) or median and interquartile range (IQR). For comparing clinical characteristics and HRQoL-scores between Study I and Study II, the McNemar test was used for categorical data and the Wilcoxon paired signed-rank test was used for continuous data. Depending on the variable, the Wilcoxon rank sum test or Fisher's Exact test was used to compare the DNHS responses between the Fontan patients and the general population. Univariate and multivariate regression analyses were performed to identify possible predictors of the PCS-12 and MCS-12 summary scores from the DNHS. To avoid type I errors due to multiple testing, a p-value of 0.01 or below was considered significant. All statistic calculations were performed using R version 4.2.0. The MatchIt-package in R was used to match Fontan patients with controls from the general population.

## Results

### Patients

A self-report version of either the PedsQL (*n* = 113) or the SF-36 (*n* = 39) was completed by 152 patients in Study I. Of these, 8 were not eligible for Study II because of: death (*n* = 4) or heart transplant (*n* = 4). Further, 35 patients were not included in Study II because of: emigration (*n* = 2), declining participation (*n* = 5), not responding to study invitation (*n* = 5), opt-out of follow-up (*n* = 1), or not completing the questionnaires (*n* = 22). This left 109 patients for final inclusion in the analyses (40 females, 69 males). Sub-analyses were conducted to check for differences between these 109 patients, and the 35 non-responders and the 8 deceased or transplanted patients, respectively. There were no significant differences in clinical characteristics or HRQoL-scores in Study I between neither of the sub-groups, when compared with the 109 patients participating in both Study I and II, please see supplementary Table 1. Of the 109 patients included in the final analyses, 74 completed the PedsQL in Study I and II, and 35 completed the SF-36 in Study I and II. Patients completing the PedsQL were significantly younger, had a lower age at Fontan completion, and a higher proportion of patients had an extracardiac tunnel Fontan type, compared to those completing the SF-36 (Supplementary Table 2). The mean time interval between Study I and Study II was 10.7 ± 1.0 years. Patient demographics for all 109 patients are described in Table 1. The mean patient age was 14.9 ± 6.6 years in Study I and 25.6 ± 6.5 years in Study II. Most patients had an extracardiac tunnel Fontan-type (60%). Tricuspid atresia (27%) and double inlet left ventricle (25%) were the most common diagnoses. The following complications increased significantly from Study I to Study II; reduced ejection fraction, atrioventricular valve regurgitation, pacemaker implantation, and documented arrhythmia (Table 2).

**Table 1:** Patient demographics

*n* = 109	*n* (%)
Sex	
Females	40 (36.7)
Males	69 (63.3)
Age, mean ± SD	
Study I	14 ± 6.6
Study II	25 ± 6.5
Ventricular morphology	
Left ventricle	70 (64.2)
Right ventricle	39 (35.8)
Diagnosis	
Tricuspid atresia	29 (26.6)
Double inlet left ventricle	26 (23.9)
Hypoplastic left heart syndrome	18 (16.5)
Unbalanced atrioventricular septal defect	13 (11.9)
Pulmonary atresia	8 (7.3)
Other univentricular hearts	15 (13.8)
Fontan type	
Extracardiac tunnel	60 (55.0)
Lateral tunnel	46 (41.2)
Classical Fontan	3 (2.8)
Age at Fontan completion	
Years (median (IQR)	3 (2.8, 5.3)

**Table 2:** Clinical characteristics in Study I and Study II

*n* = 109	Study I	Study II	
	*n* (%)		*p*
BMI (mean ± SD)	19.0 ± 4.1	23.7 ± 4.6	** < 0.001**
Echocardiography			
Ejection fraction, visually assessed			**0.003**^a^
Normal	77 (70.6)	65 (59.6)	
Mildly reduced	30 (27.5)	37 (33.9)	
Moderately reduced	1 (0.9)	4 (3.7)	
Severely reduced	1 (0.9)	3 (2.8)	
Atrioventricular valve regurgitation			** < 0.001**^b^
None	43 (39.5)	17 (15.6)	
Mild	56 (51.4)	79 (72.5)	
Moderate	9 (8.3)	12 (11.0)	
Severe	1 (0.9)	1 (0.9)	
Pacemaker	9 (8.3)	16 (14.7)	0.023
Documented arrhythmia	23 (21.1)	40 (36.7)	** < 0.001**
Medical therapy			
ACEi/ARB	24 (22.0)	26 (23.9)	0.831
Beta-blocker	9 (8.3)	16 (14.7)	0.121
ASA	61 (56.0)	65 (59.6)	0.556
Warfarin	23 (21.1)	30 (27.5)	0.146
Diuretics	19 (17.4)	23 (21.1)	0.502
Pulmonary vasodilators	0 (0)	3 (2.8)	
FALD^c^	0	7 (6.4)	
Any complication^d^	38 (34.9)	58 (53.2)	** < 0.001**
VO2_peak,_ Study II, percent predicted (%)^e^		56.6 ± 11.3	

*p*-values in bold are *p*-values below the chosen significance level of 0.05

*BMI* body mass index, kg/m^2^, *proBNP* pro–B-type natriuretic peptide, *ACEi* angiotensin-converting-enzyme inhibitors, *ARB* angiotensin receptor blocker, *ASA* acetylsalicylic acid, *FALD* fontan-associated liver disease

^a^Normal ejection fraction vs mildly/moderately/severely reduced ejection fraction

^b^No AV-regurgitation vs mildly/ moderately/ severely atrioventricular valve regurgitation

^c^There were no systematic investigations for liver disease in neither Study I nor Study II. The number of patients with FALD represents those who have presented with symptoms or findings indicative of FALD, leading to additional investigations as part of their routine clinical assessments

^d^NA in 9 patients. Percent predicted VO2_peak_ calculated with the reference equation published by Mylius et al.[19]

^e^Pacemaker, arrhythmia, ejection fraction (moderately/severely reduced), atrioventricular valve regurgitation (moderately/severe), protein-losing enteropathy, Fontan-associated liver disease

### Pediatric Quality of Life (PedsQL)

**Fig. 1:**
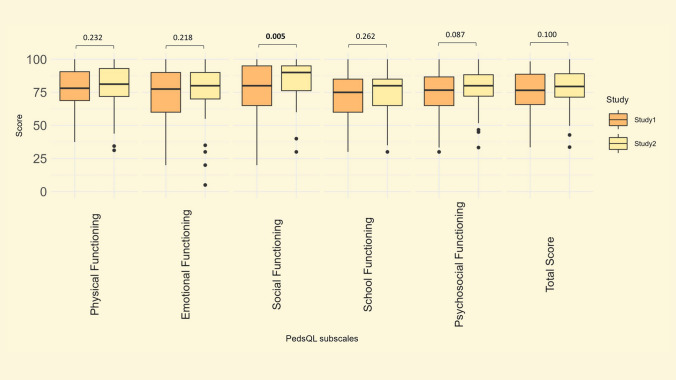
Pediatric Quality of Life (PedsQL) scores in Study I and Study II (*n* = 74). Scores range from 0 to 100, higher scores indicate better HRQoL

The PedsQL was completed by 74 patients in Study I and Study II. The mean patient age was 12 ± 3 years in Study I and 22 ± 3 years in Study II. Figure 1 presents the PedsQL scores from both studies. There was no significant difference between Study I and II in the Total Score, nor in the subscales representing Physical Functioning (median score Study I–II: 78–80), Emotional Functioning (median score Study I–II: 78–80), School Functioning (median score Study I–II: 75–80), or Psychosocial Functioning (median score Study I- II: 77–80). The only significant change was in the subscale representing Social Functioning, which increased from a median of 80 in Study I, to a median of 90 in Study II (*p* = 0.005). Looking closer at the change in PedsQL Total score from Study I to Study II, 19 patients (26%) had a decrease of 5% or more; 32 (43%) had an increase of 5% or more, while 23 (31%) had a change less than 5%. Please see Supplementary Table 3 for detailed presentation of PedsQL scores from Study I and Study II.

### 36-Item Short Form Survey (SF-36)

**Fig. 2.**
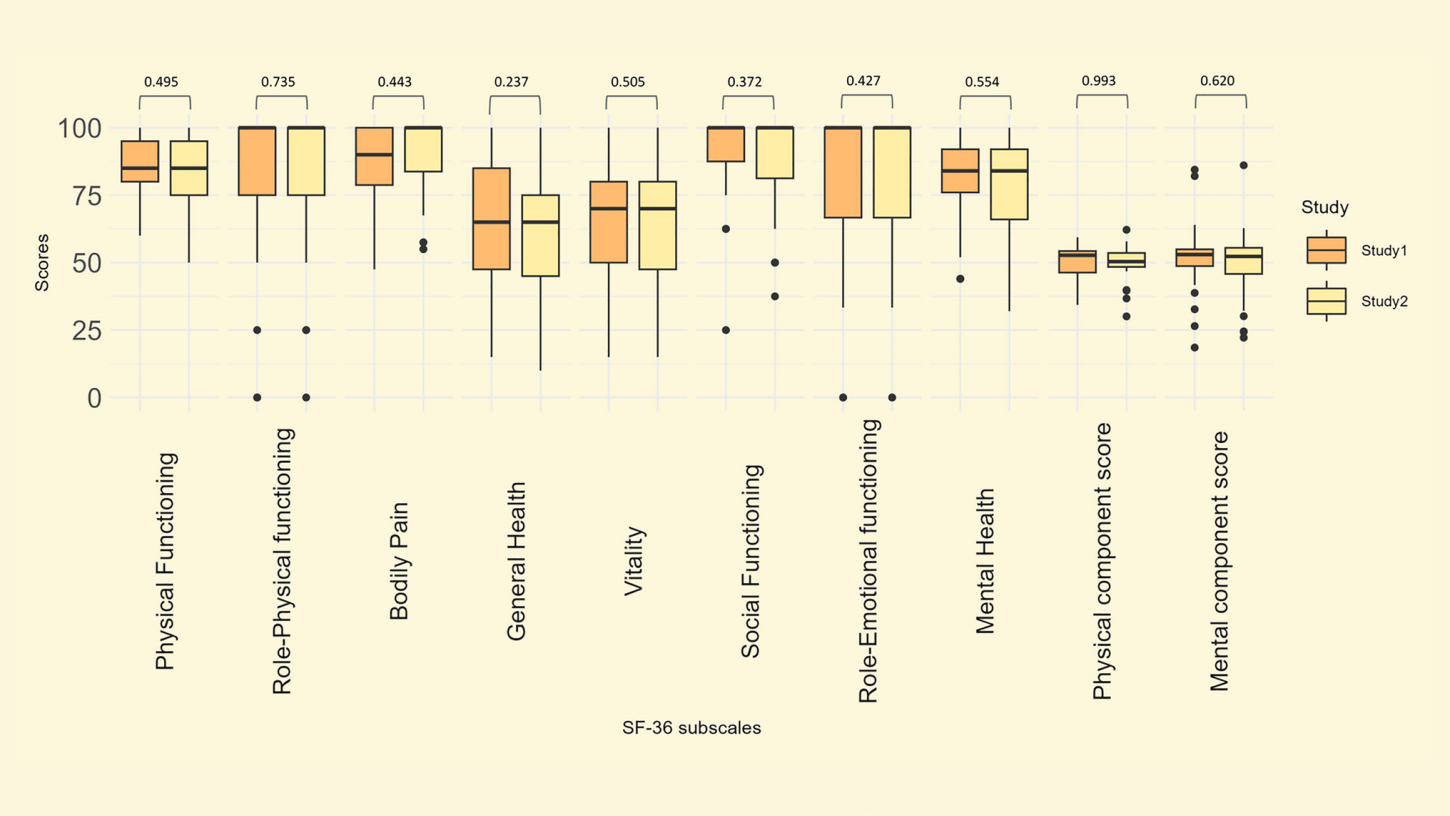
36-Item Short Form Health Survey (SF-36) scores in Study I and Study II (*n* = 35). Scores range from 0 to 100, higher scores indicate better HRQoL. The physical and mental component scores are calculated using a T-score transformation based on US normative data, to have a mean score of 50 in the US norm population, explaining the discrepancy in scores between the physical and mental component scores, and the other subscales scores

There were 35 patients who completed the SF-36 questionnaire in both studies. The mean patient age was 22 ± 6 years in Study I, and 32 ± 6 years in Study II. The scores from all subscales are presented in Fig. 2. During the study period, there were no significant changes in any of the subscales, nor in the PCS-36 or MCS-36. The Mental Health subscale had a median score of 84 in both studies. Bodily Pain had a median score of 90 in Study I and 100 in Study II, while the subscales Role-Physical, Social functioning, and Role Emotional all had a median score of 100 in both studies. The PCS-36 and MCS-36 were similar, with a median PCS-36 score of 52 in Study I and 50 in Study II, and a median MCS-36 score of 53 in both studies. Of the 35 patients completing SF-36, 8 (23%) had a decrease in PCS of 5% or more, 7 (20%) had an increase of 5% or more, while 20 (57%) had a change less than 5% from Study I to Study II. Regarding MCS, 9 (26%) had a decrease of 5% or more, 7 (20%) had an increase of 5% or more, while 19 (54%) had a change less than 5% from Study I to Study II. Please see Supplementary Table 4 for detailed presentation of SF-36 scores in Study I and II.

### The Danish National Health Survey (DNHS)

Completion of the DNHS was an inclusion criterion, and all 109 participants completed this questionnaire. By matching on sex and age, we obtained a matching ratio of 1:30 between the Fontan patients and individuals from the general population. This resulted in a control population of 3270 individuals from the general population, with the exact same age and sex distribution as the 109 Fontan patients. A comparison of demographics and DNHS responses between the Fontan patients and the general population is presented in Table 3.

**Table 3:** Comparison of the Danish National Health Survey (DNHS) between the Fontan patients and the general population

	Fontan patients (*n* = 109)	General population (*n* = 3270)	*p*
Age (median (IQR))	24 (16, 29)	24 (16, 29)	1
Sex	37.0%	37.0%	1
BMI (mean ± SD)	23.7 ± 4.6	24.4 ± 4.6	0.145
Currently smoking	15.6%	24.7%	0.040
Frequent alcohol use (4–7 days/week)	2.3%	4.7%	0.438
Highest educational level			0.019
7–9 years compulsory school	51.8%	43.4%	
Upper secondary or vocational	22.2%	17.0%	
Bachelor’s or similar	16.7%	22.0%	
Master’s or similar	5.6%	14.3%	
Other	3.7%	3.4%	
Completed higher education (bachelor’s, master’s, or similar)	22.2%	36.3%	**0.003**
Currently under education	39.4%	45.6%	0.240
Currently employed	61.1%	70.4%	0.042
General health			0.120
Excellent	11.9%	20.3%	
Very good	45.0%	45.3%	
Good	34.0%	27.2%	
Fair	7.3%	6.0%	
Poor	1.8%	1.2%	
Excellent self-rated health	11.9%	20.3%	0.029
SF-12 / HRQoL, median score (IQR)			
Physical Functioning	48 (48, 57)	57 (57, 57)	** < 0.001**
Role Physical	57 (48, 57)	57 (53, 57)	0.014
Bodily Pain	57 (47, 57)	57 (47, 57)	0.400
General Health Perception	51 (40, 51)	51 (40, 51)	0.017
Vitality	48 (38, 58)	48 (48, 58)	0.841
Social Functioning	57 (47, 57)	57 (47, 57)	0.682
Role Emotional	51 (45, 56)	56 (45, 56)	0.540
Mental Health	52 (46, 58)	52 (40, 52)	0.016
PCS-12 (physical component summary)	52 (46, 56)	56 (52, 58)	** < 0.001**
MCS-12 (mental component summary)	51 (43, 57)	50 (41, 55)	0.019
PSS (Perceived stress scale)	12 (7, 16)	12 (8, 18)	0.230
Physical activity, minutes/week, median (IQR)			
Vigorous physical activity	30 (0, 120)	120 (30, 240)	** < 0.001**
Moderate physical activity	233 (83, 420)	240 (120, 420)	0.373
Sedentary time	675 (505, 795)	660 (480, 831)	0.463
Social relations			
Infrequent contact with family	7.4%	8.0%	1
Infrequent contact with friends	7.3%	4.5%	0.164
Feeling alone	9.2%	6.9%	0.343
No one to talk to	5.5%	4.3%	0.476
Cohabitant status: lives alone	25.2%	18.3%	0.076
Cohabitant status: lives with partner	29.2%	50.0%	** < 0.001**

*p*-values in bold are *p*-values below the chosen significance level of 0.05

*BMI* body mass index, *SF-12* 12 -Item Short Form Survey, *HRQoL* health-related quality of life

Overall, there were no significant differences in educational levels. However, there was a lower percentage who had completed higher education (bachelor’s, master’s, or similar) amongst the Fontan patients compared with the general population (*p* = 0.003). Overall, general health perception was similar. Although not significant, fewer Fontan patients reported an “excellent health” (11.9%) compared with the general population (20.3%), (*p* = 0.029). The PCS-12 was significantly lower in the Fontan patients (*p* < 0.001), while the MCS-12 was similar between the Fontan patients and the background population. The median time spent on vigorous physical activity during a typical week was significantly lower in the Fontan patients (*p* < 0.001), while time spent in moderate physical activity and sedentary time was similar compared with the general population. Regarding social relations, the proportions reporting infrequent contact with family and friends were comparable between the Fontan patients and the general population. The proportions reporting problems with “no one to talk to” or “feeling alone” were also similar. However, the proportion of Fontan patients reporting to have a partner as a cohabitant, was significantly lower than in the background population (*p* < 0.001).

We conducted regression analyses using both univariate and multivariate regression models to identify possible predictors of PCS-12 and MCS-12 scores in the Fontan patients. The following variables were included in the models: sex, age, presence of any complication (arrhythmia, pacemaker, moderately or severely reduced ejection fraction, moderately or severely atrioventricular valve regurgitation, protein-losing enteropathy, Fontan-associated liver disease), percent predicted VO2_peak,_ as well as self-reported vigorous physical activity. Neither PCS-12 scores (Supplementary Table 5) nor MCS-12 scores (Supplementary Table 6) were significantly associated with any of the listed variables in either the univariate or multivariate regression models.

## Discussion

In this population-based Danish Fontan cohort of 109 patients, we found a stable HRQoL over a study period of ten years. We also compared HRQoL in the Fontan patients with the general population and found that the Fontan patients reported significantly lower physical HRQoL-scores.

### HRQoL in Fontan Patients

Several cross-sectional studies have investigated HRQoL in Fontan patients. Overall, the HRQoL scores in our patients are comparable to recent European studies in Fontan patients, both regarding PedsQL [20] and SF-36 [21]. A 2020 meta-analysis on HRQoL in Fontan patients summarized the main tendencies in previous studies well; HRQoL was significantly lower in Fontan patients compared to healthy controls across all subscales, with the most substantial score discrepancies observed in physical HRQoL [22]. Interestingly, Marshall et al. reported different results when the meta-analysis was limited to either PedsQL scores (mean patient ages 3–18 years) or SF-36 scores (mean patient ages 20–27 years). In the pooled PedsQL scores, Fontan patients had lower HRQoL across all subscales when compared to healthy peers. In the pooled SF-36 scores, only the physical and social functioning scores were significantly lower in Fontan patients, with the mental health component and mental health subscales being comparable between Fontan patients and healthy peers. These findings are comparable to results from the first HRQoL study on our cohort [12], where the Fontan patients had lower HRQoL-scores than the healthy controls in all PedsQL subscales, and in the following SF-36 subscales: Physical Functioning, General Health, and PCS-36 [12]. In Study II we observed a similar pattern: lower scores in the subscales representing physical functioning, compared to mental and social functioning. Unfortunately, we do not have PedsQL or SF-36 data from healthy controls in Study II, but the SF-12 scores from the DNHS underline the same tendency. Physical Functioning and PCS-12 were significantly lower in Fontan patients than in the general population, while there were no significant differences in either the MCS-12 or in the other subscales representing mental health or social functioning. Regarding social functioning, our results differ from the pooled SF-36 results reported by Marshall et al. Both in the SF-36 results in Study I, and in the SF-12 results in Study II, there were no significant differences in social functioning between our Fontan patients and their healthy peers. The patient cohorts included in the meta-analysis by Marshall et al. seem relatively comparable to our patient cohort regarding age, sex, and diagnoses. A possible explanation for the differing results in social functioning could therefore be attributed to external cultural and societal factors—and not related to clinical characteristics of the Fontan patients.

### Development of HRQoL Over Time

We found that the overall HRQoL in our patients remained stable during a period of ten years, regardless of the questionnaire (PedsQL or SF-36). We did not observe any decrease in the subscales in either PedsQL or SF-36. Contrary, the Social Functioning subscale in the PedsQL significantly increased (*p* = 0.005). To our knowledge, the only available reports on longitudinal HRQoL in Fontan patients are two studies conducted by the Pediatric Heart Network [7, 11]. In the 2020 study by Lambert et al., mean PedsQL scores were investigated in relation to anthropometry in Fontan patients, and comparison to our findings is not feasible [11]. In the 2017 study by Atz et al., longitudinal outcomes in the same Fontan patients are reported, including HRQoL [7]. Like us, Atz et al. reported both PedsQL and SF-36 scores and did not observe any significant changes in either of the subscales during their follow-up time of three years. The mean patient age was 18.4 ± 3.4 at the first assessment of PedsQL and SF-36, and 21.2 ± 3.5 years at the second assessment. Only selected subscale scores were presented in the study; the median PedsQL physical functioning score was stable at 78 (63, 88) in the first assessment and 75 (63, 91) in the second assessment (*p* = 0.6), while the median PedsQL psychosocial score was 73 (62, 87) in the first assessment and 77 (64, 88) in the second assessment (*p* = 0.08), which compares well to our results. The SF-36 aggregated mental and physical health scores were calculated in a different way than our results and are not directly comparable – however, both these scores also remained stable, just as the PCS-36 and MCS-36 in our results. In the same study, data from the Parent Report Child Health Questionnaire (CHQ-PF50) with a longer follow-up time of about nine years was also presented. The CHQ-PF50 was administered to Fontan patients of approximately the same age as the patients completing the PedsQL in our study (12 and 21 years in the study by Atz et al., 12 and 22 years in our study). In line with our data, Atz et al. found a stable physical summary score, and a small but significant increase in the psychosocial summary score over time; median CHQ-PF50 psychosocial summary score increased from 51 (42, 57) in the first assessment to 52 (43, 59) in the second assessment, *p* = 0.04. Although the questionnaires differ, with self-reported HRQoL in our study compared to parent-reported HRQoL in the study by Atz et al., it is interesting to find the same trend during this important phase of life.

Although the PedsQL total score did not increase significantly in our patients, 42% had an increase of 5% or more from Study I to Study II. For the SF-36 questionnaires, the majority had stable MCS-36 and PCS-36 scores displaying a less than 5% change from Study I to Study II. While the two questionnaires and the mentioned subscales are not directly comparable, the main.

difference between patients completing PedsQL and SF-36 was their age. The mean age at Study I was 12 years in those completing PedsQL, and 22 years in those completing SF-36. The increase in social functioning and the higher percentage of patients with an increase of 5% or more in HRQoL summary score among the younger patients, could imply that some HRQoL subscales are evolving in a slightly positive way for Fontan patients in this period in life. Supporting this theory, the meta-analysis by Marshall et al. found that older age at HRQoL assessment was associated with higher psychosocial scores and social functioning scores [22]. An age effect may also explain the pattern of more pronounced differences across all HRQoL subscales when comparing pediatric Fontan patients with healthy peers (using PedsQL), as opposed to only selected subscales being lower in adult patients (using SF-36) [12, 22].

One could speculate if the apparent increase in social and psychosocial HRQoL subscale scores as patients grow older reflects improved coping with the psychosocial aspects of their disease and a better understanding of their condition. Alternatively, challenges that arise during adolescence, such as heightened social expectations and an accumulation of educational and occupational life-defining choices, may pose more thought and greater difficulties in the lives of young individuals with an underlying chronic disease.

During the study period, the number of complications in our patients increased. Despite this, HRQoL did not decline – not even the physical functioning subscales. This is in accordance with other studies that investigated the effect of cardiac complications on HRQoL in Fontan patients, where no associations were found [8, 10]. Another US multicenter study found only weak associations between surgical complexity scores and HRQoL in patients with congenital heart disease (CHD) and hypothesized that other factors such as familial coping strategies may play an important role [23]. Accordingly, a systematic review by Jackson et al. concluded that lower levels of psychosocial resources in the family may give lower well-being for both the CHD patients and their families [24]. Based on this, relevant coping mechanisms among the patients and their families may explain the stability in HRQoL over time in our patients, despite the increase in complications.

It is known that patients with congenital heart disease overestimate their physical functioning in self-reported questionnaires [25]. Most of our patients perceived their health as equally good in Study II compared to Study I, even though many experienced new complications during the study period. This might reflect the discussed coping mechanisms, but it might also be the result of a well-functioning healthcare system—where complications are discovered and treated along with proper support from healthcare professionals. The disability paradox – a phenomenon where patients with persistent disabilities often report good HRQoL despite health-related challenges [26], may also explain why the HRQoL remained stable at a fairly acceptable level in a period with increases in complications. As the Fontan patients are born with their heart defect and have not experienced life in any other way, they may not perceive their HRQoL as lower than healthy people. The disability paradox phenomenon is also acknowledged in other studies investigating HRQoL in Fontan patients [22] Further investigations on this, may reveal a better understanding of the mismatch between perceived HRQoL and clinical status in Fontan patients.

### The Danish National Health Survey in Fontan Patients and the General Population

The only significant differences in HRQoL between the Fontan patients and the general population were found in the SF-12 subscales of physical functioning and PCS-12, with Fontan patients scoring significantly lower. It is reasonable that living with a Fontan circulation, which is known to give severely impaired exercise capacity in many patients [27], results in lower physical HRQoL. Despite the lower physical HRQoL in the Fontan patients, mental HRQoL, stress levels, and social relations did not seem to differ between the Fontan patients and the general population. All SF-12 subscales representing mental health were comparable between the Fontan patients and the background population. The relatively good overall HRQoL in our Fontan patients compared to the general population may be related to the above-mentioned disability paradox. As discussed in the section regarding HRQoL in Fontan patients, external factors related to the culture, society, and the healthcare system in Denmark may also play a role—and our HRQoL results may therefore not be generalizable to other parts of the world.

When comparing percentages completing all educational levels, no significant differences were found. However, a lower percentage of the Fontan patients had completed higher education, such as bachelor’s, master’s, or similar degrees, compared to the general population. These findings may be linked to the neurocognitive development challenges that many Fontan patients experience. Difficulties in cognitive functions is increasingly recognized among Fontan patients in the past decades, with genetic, fetal, and environmental factors listed as some of the possible causes [28]. Reduced cognitive speed has also been reported in our own patient cohort, in investigations conducted in relation to Study I [12]. The neurocognitive challenges found in Fontan patients could explain why some choose different educational paths than the general population. However, our patient cohort, like most Fontan cohorts, is heterogenous – and a substantial number of our patients had completed higher education. No significant difference was found in employment rates between the Fontan patients and the general population, suggesting that Fontan patients are equally active as the general population in the labor market.

The reduced physical activity levels in Fontan patients compared to healthy peers are comparable with previous findings [29]. Recent studies suggest associations between physical activity and HRQoL, making physical activity a possible target for better HRQoL [9, 30–32]. In the multivariate regression models, however, physical activity was not associated with either PCS-12 or MCS-12. Difficulties in finding predictors of HRQoL in Fontan patients are also discussed by Marshall et al. [22]. The mismatch between clinical status and HRQoL found in both our study and in previous reports, may complicate the identification of modifying HRQoL-factors. HRQoL is composed of a multitude of factors, which may make it hard to discover significant associations.

### Future Perspectives

Although our patients scored relatively good in terms of both physical and mental health over time, most cross-sectional studies find lower HRQoL in Fontan patients compared to healthy peers—especially in younger patients. Future efforts should prioritize interventions for improving HRQoL in these patients. The clinician’s recognition of reduced HRQoL among Fontan patients is important for a successful collaboration with relevant institutions, including educational and work institutions – or psychological care if necessary. The potential effect of physical activity on HRQoL may also offer promising benefits and should be further investigated.

## Limitations

Several limitations should be considered when interpreting the results of this study. First, the total number of patients of 109 is relatively small, which reduces the statistical power of the study. Furthermore, out of the 152 patients who completed Study I, only 109 were included in Study II—introducing potential selection bias. However, statistical analyses were performed to compare both the non-responders and the deceased and transplanted patients with the responders, and the groups were similar in terms of both demographics and HRQoL-scores in Study I. Unfortunately, we do not have control data for the PedsQL and SF-36 questionnaires in Study II. We do have a large number of control data from the general population in the DNHS, also containing HRQoL assessment using SF-12. We believe that control data on PedsQL and SF-36 would give similar results as with the DNHS-data, regarding differences in HRQoL between Fontan patients and the general population. Further, HRQoL is a self-reported measure, and this study can only tell how the participants report their self-perceived HRQoL when answering the questionnaires. This is a limitation in all surveys and should be accounted for when interpreting the results.

## Conclusion

We found a stable HRQoL over a period of ten years in a population-based Danish Fontan cohort. Despite an increasing occurrence of complications over the study period, both physical and mental self-reported HRQoL remained unchanged. However, the Fontan patients still reported a lower physical HRQoL than the general population.

## Supplementary Information

Below is the link to the electronic supplementary material.

Supplementary file1 (PDF 213 KB)
