# Supplementary material for: Quality of Life in the Danish Fontan Population is Unchanged Over the Past Decade—A Nationwide Longitudinal Study
Source: Pediatr Cardiol. 2023 Dec 13;46(1):148–58. doi: 10.1007/s00246-023-03347-y (PMC11753346; doi:10.1007/s00246-023-03347-y)
Supplement: Supplementary file 1 — Supplementary file1 (PDF 213 KB) [file 246_2023_3347_MOESM1_ESM.pdf]

Supplementary Table 1. Sub-group analyses comparing clinical characteristics and HRQoL scores between the 109 responders and non-responders (n=35), as well as between the 109 responders and patients who were transplanted or died during the study period (n=8). P-values represent differences from the 109 responders.

	Responders (n = 109)	Non-responders (n = 35)	p	Death or Htx (n = 8)	p
**Age**, mean ± SD	14 ± 7	17 ± 9	0.272	12± 4	0.268
**Female sex**, n (%)	40 (37)	18 (51)	0.119	3 (38)	1
**Diagnosis**, n (%)			0.808		0.129
Tricuspid atresia	29 (27)	8 (23)		0	
Double inlet left ventricle	26 (24)	8 (23)		1 (13)	
Hypoplastic left heart syndrome	18 (17)	8 (23)		5 (63)	
Unbalanced atrioventricular septal defect	13 (12)	2 (1)		0	
Pulmonary atresia	8 (7)	4 (11)		0	
Other univentricular hearts	15 (14)	5 (14)		1 (13)	
**Ventricular morphology**			0.843		0.053
Left ventricle, n (%)	70 (64)	22 (63)		2 (25)	
Right ventricle, n (%)	39 (36)	13 (37)		6 (75)	
**Fontan type**			0.246		0.081
Lateral tunnel, n (%)	60 (55)	15 (43)		7 (88)	
Extracardiac tunnel, n (%)	46 (41)	19 (54)		1 (13)	
Classic Fontan, n (%)	3 (3)	1 (3)		0	
**Age at Fontan completion**					
Years, median (IQR)	3.5 (2.7, 5.3)	3.2 (2.9, 3.7)	0.072	4.1 (3.1, 6.9)	0.839
**HRQoL Study I** [a)]					
PedsQL total score	77 (67, 88)	70 (61, 87)	0.238	66 (60, 76)	0.154
SF-36 PCS	52 (46, 54)	54 (48, 58)	0.632	-	
SF-36 MCS	53 (49, 55)	53 (51, 56)	0.439	-	

Htx (heart transplantation); HRQoL (health-related quality of life); PedsQL (Pediatric Quality of Life Inventory); SF-36 (36-Item Short Form Health Survey); PCS (physical component score); MCS (mental component score)

[a)] Of the 109 responders, 74/109 completed PedsQl and 35/109 completed SF-36 in Study I.Of the 35 non-responders, 28/35 completed PedsQl and 7/35 completed SF-36 in Study I. Of the 8 deceased or transplanted, 8/8 had completed PedsQl in Study I.

Supplementary Table 2. Comparison of baseline clinical characteristics between patients completing PedsQL or SF-36 in Study I.

	**PedsQL (n=74)**	**SF-36 (n=35)**	**p**
**Age Study I**, mean ± SD	12 ± 3	22 ± 6	**<0.001**
**Age Study II**, mean ± SD	22 ± 4	32 ± 6	**<0.001**
**Female sex**, n (%)	24 (32.4)	16 (45.7)	0.205
**Diagnosis**, n (%)			0.585
Tricuspid atresia	20 (27.0)	9 (25.7)	
Double inlet left ventricle	16 (21.6)	10 (28.6)	
Hypoplastic left heart syndrome	13 (17.6)	5 (14.3)	
Unbalanced atrioventricular septal defect	11 (12.9)	2 (5.7)	
Pulmonary atresia	6 (8.1)	2 (5.7)	
Other univentricular hearts	8 (10.8)	7 (20.0)	
**Ventricular morphology**			0.392
Left ventricle, n (%)	45 (60.8)	25 (71.4)	
Right ventricle, n (%)	29 (39.2)	10 (28.6)	
**Fontan type**			
Lateral tunnel, n (%)	26 (35.1)	20 (57.1)	**<0.001**
Extracardiac tunnel, n (%)	48 (64.9)	12 (34.3)	
Classic Fontan, n (%)	0	3 (8.6)	
**Age at Fontan completion**			
Years, median (IQR)	3.1 (2.6, 3.9)	5.9 (3.8, 10.3)	**<0.001**
**Any complication Study II** [a)], n (%)	35 (47.9)	23 (65.7)	0.100
**Percent predicted VO2peak Study II** [b)]	57.9 ± 11.0	54.0 ± 11.5	0.116

[a)] Minimum one of the following complications: documented arrhythmia; pacemaker; ejection fraction moderately or severely reduced; moderate or severe atrioventricular valve regurgitation; protein-losing enteropathy; Fontan-associated liver disease

[b)] NA in 9 patients. Percent predicted $VO2_{peak}$ is calculated with the reference equation published by Mylius et al. [1]

Supplementary Table 3. Pediatric Quality of Life (PedsQL) subscale scores in Study I and Study II, including change in scores (Δ). Scores range from 0-100, higher scores indicate better HRQoL.

**n = 74**	**PedsQL Study I**	**PedsQL Study II**	**Δ**	**P**
**Physical functioning**	78 (69, 91)	81 (72, 93)	3 (-8, 9)	0.232
**Emotional functioning**	78 (60, 90)	80 (70, 90)	0 (-10, 19)	0.218
**Social functioning**	80 (65, 95)	90 (76, 95)	5 (-4, 15)	**0.005**
**School functioning**	75 (60, 85)	80 (60, 85)	5 (-10, 20)	0.262
**Psychosocial Health Summary Score** [a)]	77 (65, 87)	80 (72, 88)	4 (-7, 18)	0.087
**Total score** [b)]	77 (67, 88)	80 (71, 89)	3 (-6, 15)	0.100

Scores are presented as median (interquartile range)

[a)] Sum of the items over the number of items answered in the Emotional, Social, and School Functioning Scales.

[b)] Sum of all the items over the number of items answered on all the Scales.

Supplementary Table 4. 36-Item Short Form Health Survey (SF-36) subscale scores in Study I and Study II, including change in scores (Δ). Scores range from 0-100, higher scores indicate better HRQoL. The physical and mental component scores are calculated using a T-score transformation based on US normative data, to have a mean score of 50 in the US norm population, explaining the discrepancy in scores between the physical and mental component scores, and the other subscales scores.

**n = 35**	**SF 36 Study I**	**SF 36 Study II**	**Δ**	**p**
**Physical functioning**	85 (80, 95)	85 (75, 95)	0 (-5, 5)	0.495
**Role-physical functioning**	100 (75, 100)	100 (75, 100)	0 (-13, 13)	0.735
**Bodily pain** (freedom from)	90 (79, 100)	100 (90, 100)	0 (-6, 6)	0.443
**General health**	65 (48, 85)	65 (45, 75)	-10 (-20, 13)	0.237
**Vitality**	70 (50, 80)	70 (50, 80)	-5 (-15, 10)	0.505
**Social functioning**	100 (88, 100)	100 (81, 100)	0 (-6, 0)	0.372
**Role-emotional functioning**	100 (67, 100)	100 (67, 100)	0 (0, 33)	0.427
**Mental health**	84 (76, 92)	84 (66, 92)	0 (-8, 4)	0.554
**Physical component score**	52 (46, 54)	50 (48, 54)	0 (-4, 4)	0.993
**Mental component score**	53 (49, 55)	53 (46, 56)	0 (-6, 4)	0.620

Supplementary Table 5. Regression analyses investigating possible predictors of the SF-12 physical component score (PCS-12).

n = 109	Univariate		Multivariate	
**Variable**	**Estimate**	**p**	**Estimate**	**p**
**Sex** (reference = male)	-3.6 ± 1.4	0.011	- 3.7 ± 1.5	0.014
**Age**	- 0.2 ± 0.1	0.033	- 0.1 ± 0.1	0.208
**Any complication** [a] (reference = no complications)	-2.9 ± 1.4	0.037	- 2.3 ± 1.5	0.126
**Self-reported vigorous physical activity** (reference = no vigorous physical activity)				
10 – 119 minutes/week	- 3.7 ± 1.8	0.043	- 3.7 ± 1.8	0.042
≥ 120 minutes/week	1.9 ± 1.6	0.258	- 0.2 ± 1.8	0.896
**VO2peak, percent predicted** [b]	0.2 ± 0.1	0.015	0.1 ± 0.1	0.105

[a] Minimum one of the following complications: documented arrhythmia; pacemaker; ejection fraction moderately or severely reduced; moderate or severe atrioventricular valve regurgitation; protein-losing enteropathy; Fontan-associated liver disease

[b] NA in 9 patients. Percent predicted $VO2_{peak}$ is calculated with the reference equation published by Mylius et al. [1]

Supplementary Table 6. Regression analyses investigating possible predictors of the SF-12 mental component score (MCS-12).

N = 109	Univariate		Multivariate	
**Variable**	**Estimate**	**p**	**Estimate**	**p**
**Sex** (reference = male)	-3.2 ± 2.2	0.139	- 3.6 ± 2.2	0.109
**Age**	- 0.0 ± 0.2	0.815	- 0.1 ± 0.2	0.444
**Any complication** [a] (reference = no complications)	- 1.8 ± 2.1	0.383	- 2.9 ± 2.2	0.197
**Self-reported vigorous physical activity** (reference = no vigorous physical activity)				
10 – 119 minutes/week	- 1.3 ± 2.5	0.603	- 0.4 ± 2.6	0.885
≥ 120 minutes /week	-0.1 ± 2.3	0.955	- 0.7 ± 2.7	0.796
**VO2peak, percent predicted** [b]	0.0 ± 0.1	0.976	- 0.0 ± 0.1	0.790

[a] Minimum one of the following complications: documented arrhythmia; pacemaker; ejection fraction moderately or severely reduced; moderate or severe atrioventricular valve regurgitation; protein-losing enteropathy; Fontan-associated liver disease

[b] NA in 9 patients. Percent predicted $VO2_{peak}$ calculated with the reference equation published by Mylius et al. [1]

[1] C. F. Mylius *et al.*, "Peak oxygen uptake reference values for cycle ergometry for the healthy dutch population: Data from the lowlands fitness registry," *ERJ Open Res*, vol. 5, no. 2, 2019, doi: 10.1183/23120541.00056-2018.
